# The fate of volcanic ash: premature or delayed sedimentation?

**DOI:** 10.1038/s41467-021-21568-8

**Published:** 2021-02-26

**Authors:** Eduardo Rossi, Gholamhossein Bagheri, Frances Beckett, Costanza Bonadonna

**Affiliations:** 1 Department of Earth Sciences, University of Geneva, Rue des Maraichers 13, Geneva, Switzerland; 2 Max Planck Inst. Dynam. & Self Org., Fassberg 17, Gottingen, Germany; 3 Met Office, Fitzroy Road, Exeter, UK

**Keywords:** Natural hazards, Volcanology

## Abstract

A large amount of volcanic ash produced during explosive volcanic eruptions has been found to sediment as aggregates of various types that typically reduce the associated residence time in the atmosphere (i.e., premature sedimentation). Nonetheless, speculations exist in the literature that aggregation has the potential to also delay particle sedimentation (rafting effect) even though it has been considered unlikely so far. Here, we present the first theoretical description of rafting that demonstrates how delayed sedimentation may not only occur but is probably more common than previously thought. The fate of volcanic ash is here quantified for all kind of observed aggregates. As an application to the case study of the 2010 eruption of Eyjafjallajökull volcano (Iceland), we also show how rafting can theoretically increase the travel distances of particles between 138–710 μm. These findings have fundamental implications for hazard assessment of volcanic ash dispersal as well as for weather modeling.

## Abstract (web summary)

Generally it is thought that ash aggregation leads to reduced atmospheric travel distances. Here, the authors show that the rafting effect can increase dispersal range by up to 3.7 times for particles between 300–500 μm, compared to sedimentation of individual clasts.

## Introduction

**Fig. 1:**
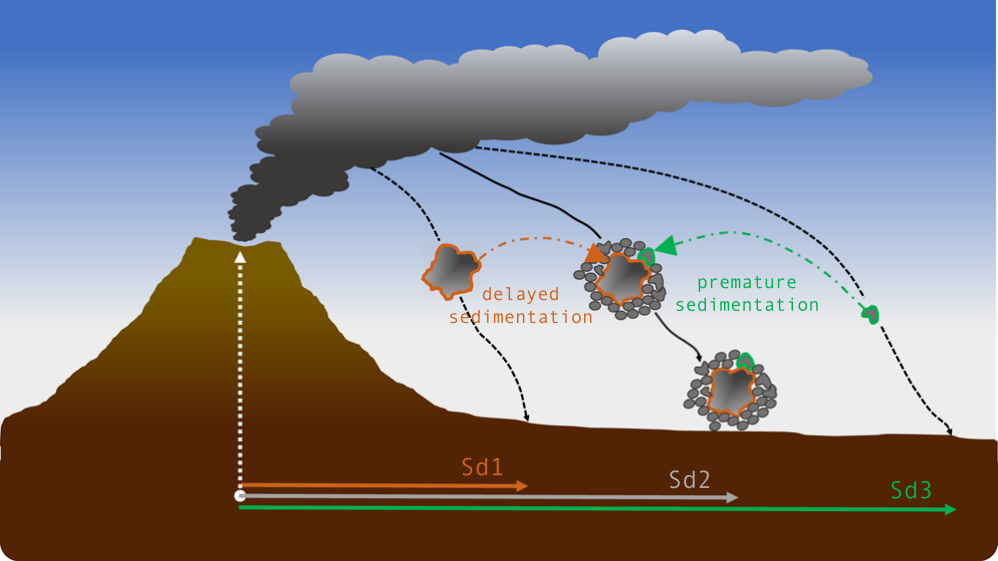
Premature and delayed sedimentation. Simplified sketch showing both premature and delayed sedimentation of single particles due to aggregation. Premature sedimentation of a single particle (typically <63 microns) occurs when it falls closer to the volcano (at distance Sd2) than it would do if not part of an aggregate (distance Sd3). On the contrary, a particle that acts as an aggregate core in a cored cluster could be rafted and sediment at larger distance from vent (Sd2) than if it was falling individually (distance Sd1).

Sedimentation of volcanic ash needs to be accurately described both for real-time forecasting of atmospheric dispersal and long-term hazard assessment of ground tephra loading in order to reduce risk associated with explosive volcanic eruptions^1,2^. The atmospheric transport of volcanic particles with diameter >20 µm is also crucial to weather modeling as they are believed to have a major role in several weather processes (e.g., rain nucleation)^3,4^. Nonetheless, the fate of volcanic ash is still not fully understood and constrained as the associated aerodynamics and residence time in the atmosphere are controlled by complex sedimentation processes^5^. As an example, the clustering of volcanic ash (i.e., particle aggregation) is commonly believed to affect the sedimentation of the tephra fraction <63 μm (i.e., fine ash) by reducing its residence time from days or weeks to less than one day^6^. However, we explore here how aggregation may not always reduce the residence time of volcanic ash in the atmosphere but, in some cases, may raft coarse ash (i.e., ash comprised between 63 μm and 2000 μm) acting as aggregate cores to larger distances from the volcano than expected (Fig. 1).

Various evidence exists of unexpected large sedimentation distances of particles between 100–500 μm based on direct observations and geophysical monitoring that have been so far related to particle shape or ice coating^7–14^. Particle rafting could also be an alternative explanation; however, previous studies^15,16^, which calculated particle drag assuming Stokes flow, concluded that even though particle rafting was a potential result of aggregation, it was most unlikely. Nonetheless, the use of Stokes’ drag is only justified for low particle Reynolds number *Re* (i.e. for particles and aggregates smaller than about 50 μm), while most aggregates sediment at higher Reynolds numbers. New insights from field observations suggest that rafting is not only possible but probably more frequent than previously thought^17^. In fact, a strong link has been identified between rafting and cored clusters, a specific typology of aggregates also defined as Particle Clusters Type 3 (PC3)^17^ to be consistent with the current classification used in literature^18^. Cored clusters indicate coarse-ash particles (≈200–1000 μm)—cores—that are coated by a thick layer (≈100–300 μm) of smaller particles (<80 μm) with a density typically <1500 kg m^−3^ ^17,19^. As for all other Particle Clusters identified so far (i.e., PC1 and PC2)^18^, cored clusters (PC3) are difficult to observe or collect due to the low preservation potential in deposits. The optimal strategy to characterize PC3 is based on a combined use of high-speed footage and adhesive paper compatible with Scanning Electron Microscopy^17,19^.

In this paper we quantitatively investigate under which circumstances ash aggregation results in a simultaneous premature sedimentation of fine ash and delayed sedimentation of coarse ash. We demonstrate that rafting may occur under specific conditions due to the non-linear behavior between weight and drag forces acting on aggregates when falling through the atmosphere. This information is summarized in dedicated sedimentation charts (described in section 2.1), a powerful tool to investigate the fate of volcanic ash in the atmosphere. In this work we found that the lower density that usually characterizes aggregates with respect to single particles, is not sufficient to delay the sedimentation of coarse ash in the atmosphere. A specific packing configuration (i.e., aggregate porosity *ϕ*_*A*_ and aggregate-to-core size ratio Λ) is required to trigger particle rafting. Under this condition, the concurrent presence of fine and coarse ash in a given aggregate has the potential to simultaneously reduce the residence time of fine ash in the atmosphere and increase the sedimentation distance of coarse ash. Thus, the transport capacity of fine ash in volcanic aggregates is closely affected by the presence of coarse ash, and vice versa. The main objective of this paper is to identify the theoretical boundaries under which premature or delayed sedimentation of volcanic ash occurs during tephra fallout and how this affects the transport of volcanic ash in the atmosphere.

## Results

### The sedimentation charts: when is the sedimentation of a single particle accelerated or delayed?

The dynamics of a falling ash aggregate depends on the balance between the competing forces of drag and weight. On the one hand, the aggregate is of greater mass than its constituting particles, which leads to premature deposition of the fine-ash fraction (i.e., the aggregate shell). On the other hand, the increase in diameter and the change in shape associated with the physical process of clustering may augment the drag force, with the potential to delay the sedimentation of the aggregate core (Fig. 1).

**Fig. 2:**
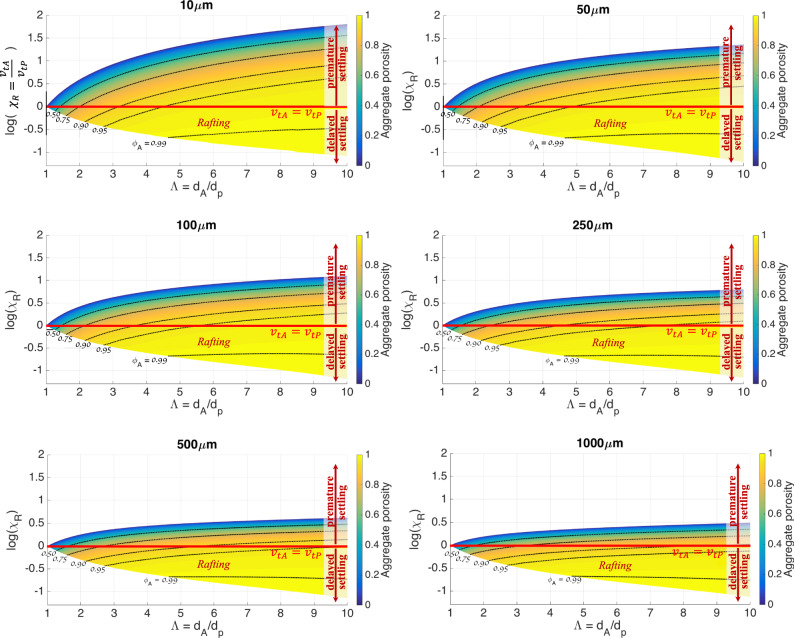
The sedimentation charts. Premature and delayed sedimentation expressed by the rafting factor (*χ*_*R*_) as a function of the aggregate-to-core diameter ratio (*Λ*) and of the aggregate porosity (*ϕ*_*A*_) for six different core sizes (10 μm, 50 μm, 100 μm, 250 μm, 500 μm, 1000 μm). The red line *v*_*tA*_ = *v*_*tP*_ indicates the separation between premature (log(*χ*_*R*_) > 0) and delayed (log(*χ*_*R*_) < 0) sedimentation.

Premature and delayed sedimentation of a given ash particle can be quantitatively defined in terms of the rafting factor $\chi _R = \frac{{v_{tA}}}{{v_{tP}}},$ which describes the ratio between the terminal velocity of the aggregate, *v*_*tA*_, and the terminal velocity of an individual particle inside the aggregate (typically the core), *v*_*tP*_ (Fig. 2, i.e., sedimentation charts). If *χ*_*R*_ > 1, the considered particle is prematurely sedimented (i.e., log(*χ*_*R*_) > 0 in Fig. 2), while sedimentation is delayed if *χ*_*R*_ < 1 (i.e., log(*χ*_*R*_) < 0 in Fig. 2). The calculation of *χ*_*R*_ is straightforward once the characteristics of the single particles and of the aggregate are established, i.e., *d*_*P*_, *ρ*_*P*_ and *d*_*A*_, *ϕ*_*A*_, respectively (see methods). Finally, for a fixed particle of size *d*_*P*_, the parameter *χ*_*R*_ can be conveniently expressed as a function of the aggregate porosity *ϕ*_*A*_ and the aggregate-to-core size ratio, ${{\Lambda }} = \frac{{d_A}}{{d_P}}$.

It is worth noticing that the new sedimentation charts computed in the present work (Fig. 2) show wider regions for rafting if compared to Stokes’ drag formula (Supplementary Fig. 2), which explains why rafting has been considered unlikely so far from a theoretical point of view^15^. As a matter of fact, spherical particles can be adequately described in terms of Stokes’ drag $C_D^{ST}$ only for *Re* less than ≈1; for larger values of *Re*, as expected for the sedimentation of volcanic ash larger than 50 μm, a correct expression for the drag coefficient is required both for spherical and non-spherical objects^20^, referred to here as $C_D^{BB}$ (see methods). In summary, it is the use of a more generalized drag term such as $C_D^{BB}$ in the drag force instead of $C_D^{ST}$that makes rafting more likely to occur in nature than thought so far.

### Premature or delayed sedimentation of most typical aggregate types

**Fig. 3:**
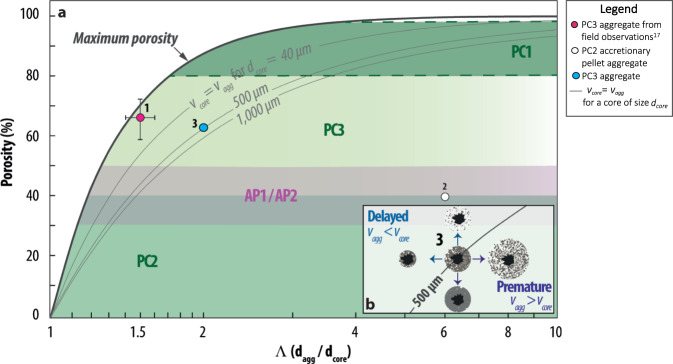
Aggregate types and rafting. Generalized plot showing the potential for particle rafting for different types of aggregates. **a** Green zones indicate observed values of porosity (*ϕ*_*A*_) for PC1^18, 19^, PC2^19^ and PC3^17, 19^, while the pink area indicates the typical values of porosity for AP1 and AP2^18, 43^ (see also table 16.1 in Sparks et al. 1997^44^). Unfortunately, the few studies present in literature for PC1 do not allow a good constraint on the values of the associated porosities (indicated, therefore, as dashed lines). Aggregates 1 and 2 represent a PC3 from Sakurajima 2013 Vulcanian eruption^17^ and a well-structured accretionary pellet (AP2) from the 26 December 1997 dome collapse of Soufrière Hills volcano, Montserrat, respectively;^21^ aggregate 3 represents a generic PC3 with core size of 500 μm (with characteristics observed during the 2013 Sakurajima eruption^17^). The errorbar associated with aggregate 1 represents the 68% of uncertainty on *Λ* and *ϕ*_*A*_ respectively. **b**) A zoom around aggregate 3 to show the influence of aggregate porosity on premature and delayed sedimentation of the core; in all cases the particles of the shell are prematurely sedimented. The premature sedimentation of the coating ash can be seen considering each single particle as a fictitious core characterized by large values of *Λ* (*Λ* ≫ 10): Fig. 3 shows that ash characterized by *Λ* ≫ 10 and size *d*_*P*_ ≲ 100 μm is not subjected to rafting.

The theoretical concepts expressed in Fig. 2 are here applied to the existing classification of volcanic aggregates^17,18^. In particular, Fig. 2 is rearranged to simultaneously display the parameters Λ and *ϕ*_*A*_ together with the typical features of observed aggregates available in literature (Fig. 3). Equal velocity curves of *χ*_*R*_ = 1 (i.e., equal terminal velocity of aggregate and core) are plotted for aggregates with core diameters of 40, 500 and 1000 μm, respectively.

Due to mass conservation, the porosity of an aggregate cannot be above the maximum porosity curve, which corresponds to 100% porosity of the shell (Eq. 1.7–Eq. 1.9 in Supplementary Note 1). Aggregate 1 represents a cored cluster observed at Sakurajima^17^ that has a core diameter of ~500 μm, Λ ≈ 1.5 and *ϕ*_*A*_ ≈ 68% and, therefore, lies above the corresponding equal velocity curve; resulting *χ*_*R*_ is ≈ 0.68 − 0.77 (i.e., delayed sedimentation of aggregate core). In contrast, aggregate 2 represents a Pellet with Concentric Structure (AP2; equivalent to Accretionary Lapilli) as observed for the 26 December 1997 dome collapse of Soufrière Hills volcano, Montserrat^21^, with a core diameter of 1000 μm and porosity of 40% that falls below all equal velocity curves; the resulting settling velocity is ≈2 times larger than that of an individual particle of 1000 μm (i.e., premature sedimentation of all aggregate components). Analyzing a generic PC3 (i.e. aggregate 3 in Fig. 3a) we can see how premature or delayed sedimentation depends on the associated values of porosity *ϕ*_*A*_ and ${{\Lambda }} = \frac{{d_A}}{{d_P}}$ (Fig. 3b). In this specific case, the core particle has a diameter of 500 μm, a porosity of 63% and a Λ of 2 (i.e., the whole aggregate has a diameter of 1000 μm). The aggregate core is rafted in case of an increase in porosity and/or a decrease in Λ (i.e., when the aggregate is above the line of *χ*_*R*_ = 1); in contrast, all particles are prematurely sedimented in case of a decrease in porosity and/or an increase in Λ (i.e. when the aggregate is below the line of *χ*_*R*_ = 1).

This analysis suggests that Pellets with Concentric Structure Pellets (AP2;^18^ most commonly defined as Accretionary Lapilli) are typically associated with premature fallout of all particles (given that most of the region compatible with this aggregate type is below all lines of *χ*_*R*_ = 1), while Particle Clusters can be associated with both premature or delayed fallout of the aggregate core depending on specific values of porosity (*ϕ*_*A*_) and aggregate-to-core size ratio (Λ). It is important to stress that even though the aggregate core can be rafted, the surrounding shell is always associated with premature sedimentation. In fact, fine ash in the shell can be modeled as a fictitious core with large values of Λ (i.e., Λ ≫ 10), for which rafting is unlikely to occur (Fig. 2).

**Fig. 4:**
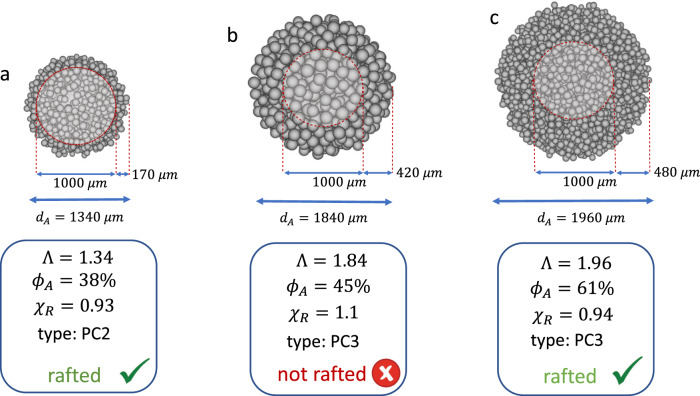
Examples of rafted and not rafted aggregates. PC2 and PC3 aggregates associated with a core of 1000 *μ*m, generated with SCARLET-1.0^22^, a dedicated software for virtual reconstruction of aggregates. Aggregate type (e.g., PC2 or PC3) is defined according to the combinations of *ϕ*_*A*_ and Λ reported in Fig. 3 and in the literature^18^. In detail: **a** PC2 aggregate with a coating made of 1000 particles of sizes between 70 μm and 90 μm; the resulting porosity *ϕ*_*A*_ and aggregate-to-core size ratio Λ generate a delayed sedimentation of the core (*χ*_*R*_ < 1). **b** PC3 aggregate with a coating made of 1000 particles between 120 μm and 150 μm that generate a premature sedimentation of the core (*χ*_*R*_ > 1). **c** PC3 aggregate with a coating made of 4000 particles of sizes between 70 μm and 90 μm that result in a delayed sedimentation of the core (*χ*_*R*_ < 1).

A key message of Fig. 3 is that rafting of the aggregate core can occur even at relatively low porosities (*ϕ*_*A*_ ≳ 20 − 30%), if the aggregate-to-core ratio Λ is ≲ 2 and the core size is larger than ≈200 μm. As an example, by means of a dedicated software^22^, we simulated three aggregates that, according to their combinations of *ϕ*_*A*_ and Λ (see Fig. 3), are classified respectively as a PC2 (Fig. 4a) and PC3 aggregates (Fig. 4b, c), but with different porosities. In all of these cases the core has a size *d*_*P*_ = 1000 μm. In the PC2 case, the coating is composed of 1000 particles between 70–90 μm in size that are arranged to give a bulk porosity *ϕ*_*A*_ = 38% and an aggregate-to-core ratio Λ = 1.34 (Fig. 4a). This configuration is already sufficient to produce a delayed sedimentation of the core (rafting factor of *χ*_*R*_ = 0.93). However, larger values of Λ rapidly lead to an increase of the mass of the shell, which then requires higher porosities in order for the core to be rafted. As an example, the PC3 aggregate of Fig. 4b is made of 1000 particles between 120–150 μm that are arranged in such a way that the increase of the gravitational force due to the additional mass overcomes the effects of the increase drag (*ϕ*_*A*_ = 38%, Λ = 1.84) resulting in a premature sedimentation of the core (*χ*_*R*_ = 1.1). Nonetheless, rafting can be obtained for a PC3 aggregate if a looser configuration of the coating makes the drag predominant with respect to gravity. This is the case of the PC3 of Fig. 4c, where 4000 particles between 70–90 μm produce a porosity *ϕ*_*A*_ = 61% and a size ratio Λ = 1.96 (*χ*_*R*_ = 0.94). It is the arrangement of particles in the coating shell which play the key role for rafting, and this can only be triggered if the increase in mass occurs at the expenses of a rapid increase in the external surface of the aggregate (e.g., increasing its porosity). As a conclusion, premature or delayed sedimentation should be seen as a consequence of the combined effect of *ϕ*_*A*_ and Λ, and not as a result of their individual impact.

### Quantification of travel distances: the case of the 2010 Eyjafjallajökull eruption

The impact of aggregation on the transport of volcanic ash is investigated for the 2010 eruption of Eyjafjallajökull volcano (Iceland) together with the influence of particle shape. We consider eruption and atmospheric conditions associated with the period between 5–8 May 2010 and properties of particles and aggregates observed on 5 May 2010 at 9.7 km from vent^23^ (densities, equivalent diameter, elongation, and flatness are reported in Supplementary Note 4). We model the atmospheric transport of both individual particles and aggregates using NAME (Numerical Atmospheric dispersion Modeling Environment)^24^ with meteorological data from the Global configuration of the U.K. Met. Office Unified Model^25^. The model setup is given in Table 1. The maximum travel distance of a particle/aggregate (of a given size, shape and density) was determined by calculating the great-circle-distance at which 95% of its total erupted mass had been deposited.

**Table 1:** Input parameters for the NAME runs.

Model Parameter	Value
Source Location	Eyjafjallajökull, 63.63° lat, −19.62° lon
Vent Height	1666 m above sea level
Source Start + End Times	00:00 04/05/2010 – 00:00 09/05/2010
Source Shape	Line source, from vent to plume top, uniform distribution
Plume Height	5,500–10,000 m asl^26^
Source Strength	1 × 10^6^ g hr^−1^ (Unit Release)
Model Particle Release Rate	1500 hr^−1^
Meteorological Data	Unified Model (Global Configuration) 25 km horizontal resolution (midlatitudes), 3 Hourly Temporal Resolution

Note that the source strength and model particle release rate relate to simulations of each particle/aggregate (i.e. for each size, shape, density combination considered).

We performed a series of simulations which considered the transport of different particles and aggregates of varying size, density and shape. We considered sizes <1500 μm which guarantees a particle relaxation time on the order of a few seconds, appropriate for consideration in NAME^24,26^, and consistent with operational forecasting of the long-range transport of ash clouds^27^. For the two most relevant size bins (phi = 3 and phi = 2), we considered the median of the equivalent diameters as representative of the entire size range, with the equivalent diameter defined as *d*_*eq*_ = (*L*∙*I*∙*S*)^1/3^. The resulting median values of the equivalent diameters, i.e., $d_{eq}^{phi = 3} = 138\;$μm and $d_{eq}^{phi = 2} = 447\;$μm, are used as the inner core sizes for the released aggregates, for which the shape have been observed^23^ and the density can be derived from the size-density calibration curve reported in the literature^28^. The resulting core densities are 2039 kg m^−3^ for $d_{eq}^{phi = 3}$ and 1864 kg m^−3^ for $d_{eq}^{phi = 2}$ (Supplementary Tables 2 and 3). Each combination of porosity and aggregate-to-core ratio assures that *ϕ*_*A*_ values do not exceed the maximum theoretical value allowed for a given Λ (Eq. 1.8 in Supplementary Note 1). We computed the maximum travel distances of (i) the core particles with their actual shape and density; (ii) the core particles assumed as spheres of equivalent diameter; (iii) spherical aggregates with various porosities (*ϕ*_*A*_ from 40% up to the maximum observed value of *ϕ*_*A*_ = 90% reported in literature^18^) and various aggregate-to-core ratios (Λ = 1.5, 2, 5), calculated relatively to the specific core size under analysis. The densities of the aggregates are calculated from the porosity *ϕ*_*A*_ and the core density as indicated in Eq. (4) (see methods). The values of *ϕ*_*A*_ are consistent with field observations^17,19,29^ and laboratory investigations^16,30^, which suggest how electrostatic charges or microscopic water layers can act as main binding mechanisms, resulting in a wide range of porosities according to the relative size of the particles involved in the collision processes.

**Fig. 5:**
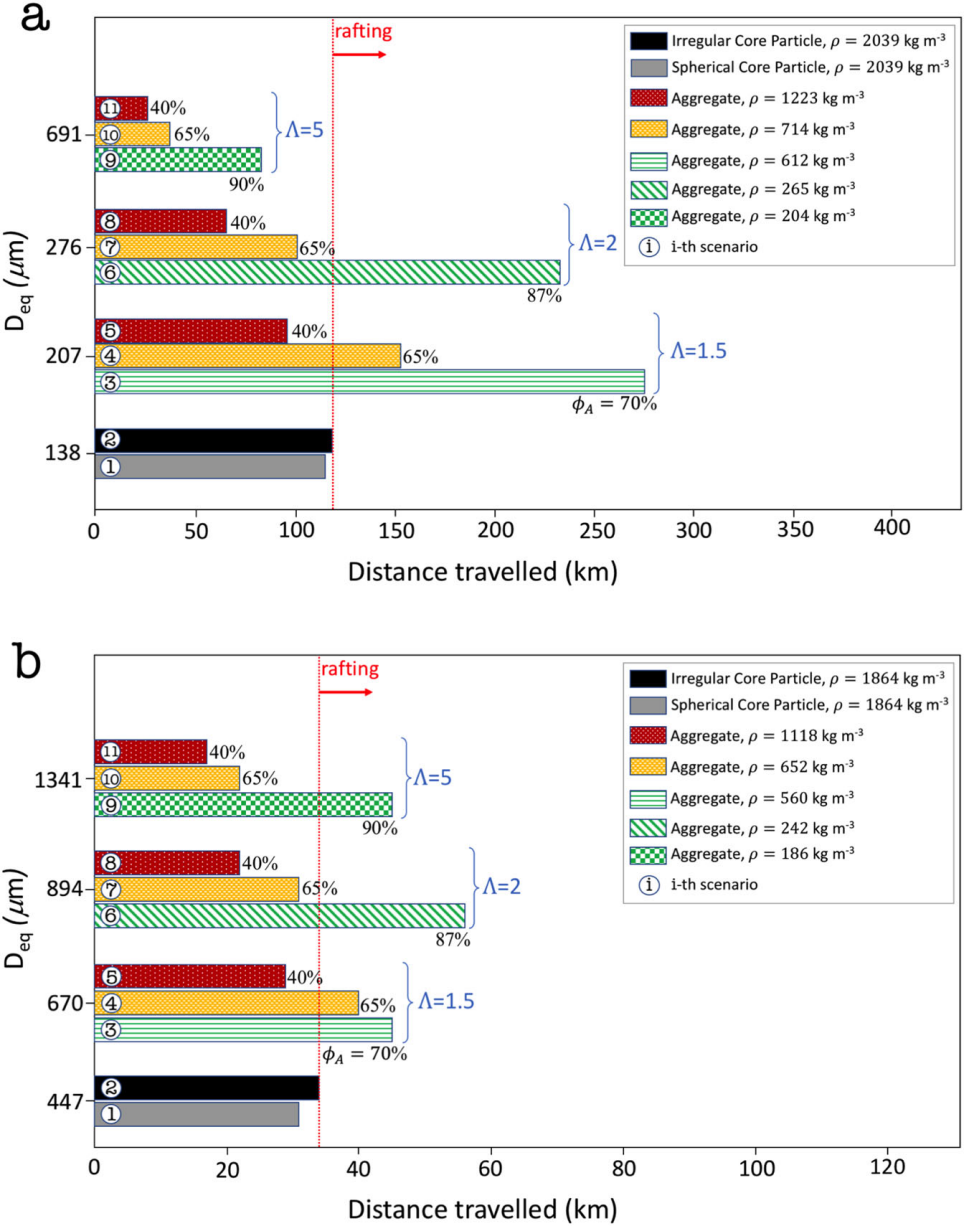
Premature or delayed sedimentation during the 2010 Eyjafjallajökull eruption. Sedimentation distances traveled by core particles of 138 μm (**a**) and 447 μm (**b**) when they are considered as single objects, either spherical or with their observed shape^23^, or when they act as a core for aggregates with different size ratios *Λ* and porosities *ϕ*_*A*_ (note different x-axis scales); particles of 138 μm and 447 μm correspond to the median of the equivalent diameters *d*_*eq*_ = (*L I S*)^1/3^ of all the objects belonging respectively to the bins phi = 3 and phi = 2. Particle shape reflects observations for the Eyjafjallajökull ash^23^. The median value is used as supposed to the arithmetic mean to guarantee that both the diameters and the shapes used in the simulations were really observed during the eruption. Reported travel distances represent the great circle distance from the vent. The maximum travel distance of a particle/aggregate is defined as the point at which 95% of its erupted mass had been deposited.

As expected, the travel distance of the individual core particles decreases with increasing diameters, with irregular particles traveling 1.02 and 1.10 times further with respect to spherical ones, for cores with size 138 μm and 447 μm, respectively (2% and 10% increase in travel distance, respectively; Fig. 5). However, when considering the travel distance of the core particles as part of aggregates, the scenario can vary significantly depending on the aggregate size and porosity. The core particles investigated can be rafted and travel from a minimum of 1.3 times up to a maximum of 2.7 times further with respect to individual spherical particles (i.e. 30% and 170% increase in travel distance, respectively) and between 1.2 times and 2.2 times with respect to irregular particles (i.e. 20% and 120% increase in travel distance, respectively) (scenarios 3, 4 and 6 in Fig. 5a and scenarios and 3, 4, 6 and 9 in Fig. 5b). Delayed sedimentation is more likely to occur for a wide range of porosity and aggregate-to-core ratio (Λ) as the inner core size increases. As an example, the 447 μm core particle is rafted for porosities >47% and all aggregate-to-core ratio values investigated (1.5–5). Even though the 138 μm core particle is rafted for fewer scenarios investigated (i.e., porosity >53% but only for aggregate-to-core ratio values of 1.5–2), the increase in travel distance due to rafting is larger (20–120%; scenarios 3, 4 and 6 in Fig. 5b). Where the two competitive processes of increasing mass and drag balance each other, the travel distance of the aggregate equals the travel distance of the spherical core particle (e.g., scenario 7 in Fig. 5b). The effect of the shape also becomes relatively more important as the size increases, as clearly evident comparing scenarios 1 and 2 for Fig. 5a, b.

## Discussion

Our work provides the first theoretical constraint of particle rafting and shows how aggregation can simultaneously cause premature fallout of fine ash and delayed sedimentation of the associated aggregate cores (typically in the coarse-ash size range). We demonstrate that specific packing configurations of fine and coarse ash inside an aggregate produce values of porosity (*ϕ*_*A*_) and aggregate-to-core size ratio (Λ) that reduce the settling velocity of coarse ash and increase the settling velocity of the fine ash that forms the coating around the core. The transport capacity of coarse ash in the atmosphere is, thus, closely related to the presence of fine ash in the volcanic plume and cloud, and vice versa. The sedimentation charts of Fig. 2 show that the fate of aggregating particles in their path across the volcanic plume/cloud and the atmosphere is strongly dependent on the size and relative abundances of the other particles present in the aggregate. Ultimately, premature or delayed sedimentation is a direct consequence of how a given porosity (*ϕ*_*A*_) is reached at the expenses of the aggregate-to-core size ratio (Λ).

The theoretical framework introduced in the present work combined with field^17,19,29^ and laboratory observations^16,30^ allows some speculations on the connection between binding mechanisms inside volcanic ash aggregates and rafting. The description of particle rafting in terms of aggregate types and structure (Figs. 3 and 4) suggest that some binding mechanisms are more likely to result in a delayed sedimentation than others. According to the existing literature, the main binding mechanisms are electrostatic forces^16,30,31^, dissipation due to viscous forces in water layers^32^, ice formation;^33^ and salt bridges due to mineral precipitation^34,35^. The experiments of James et al.^16,30^. demonstrated that electrostatic forces can produce aggregates characterized by high porosities in the shell, a condition that promotes delayed sedimentation if combined with small aggregate-to-core ratios (Λ). The fact that rafting has been observed at Sakurajima Volcano^17^, where previous studies already proved the presence of high electrification within ash aggregates^32^, supports the role of electrostatic forces in promoting delayed sedimentation of coarse ash. Nevertheless, this consideration cannot exclude that other binding mechanisms, such as the presence of water films or salt bridges, may produce appropriate combinations of Λ and *ϕ*_*A*_ in the shell that finally trigger particle rafting. However, the high values of aggregate-to-core ratios (Λ > 2) and the relatively low maximum porosities ($\phi _A^{Max} \approx 50\%$) that characterize Well-Structured Accretionary Pellets (AP2; Accretionary Lapilli) suggest that the binding mechanisms involved, such as a macroscopic water phase and salt precipitation, can more likely induce premature sedimentation.

Our results can be applied to any type of aggregate, as long as the size and densities of the objects involved in the aggregation process are known. As an example, if dis-aggregation or new aggregations occur during the transport, the resulting products will still be described by the sedimentation charts (Fig. 2) or our generalized sketch (Fig. 3). The application of the sedimentation charts to the 2010 eruption of Eyjafjallajökull volcano (Iceland) show how aggregate cores can be rafted a few to hundreds of kilometers further from the volcano than if they sedimented as individual particles. In particular, we have shown how rafting can cause larger travel distances with respect to the effect of increase of particle drag of irregular particles compared to the assumption of sphericity. Nonetheless, our results of Fig. 5 are specific to one eruption; more investigations on the effect of increase of drag due to both particle aggregation and particle shape should be carried out for a larger dataset of eruptions and particle characteristics (shape, density) to better characterize the increase in travel distance due to these two aspects. In addition, travel distances of larger core particles (>500 μm) should also be investigated; here we only explored the range of travel distances of particles typically considered in operational modeling using NAME^26,27^ (i.e. particles with Stokes number <1). Finally, observations of large particles far from the source and their associated relative mass are not currently captured in satellite retrievals and should be better constrained based on a concerted effort to improve remote sensing^7,8,13^.

We can also conclude that in modeling dispersal and deposition of volcanic ash, aggregation processes and physical characteristics (i.e., size, density and shape) of aggregates and single particles need to be accurately described. When these physical characteristics are accounted for in the calculation of their fall velocity, we find that ash can travel much further than single spherical particles of the same size. Previous studies have generally not considered the effect of density when modeling the transport of both non-aggregated^36,37^ and aggregated^38–40^ volcanic ash and has often assumed as a constant value. However, our analyses show that density of both individual particles and aggregates represents a critical parameter that needs to be well constrained in order to accurately describe the dispersal and sedimentation of ash when aggregation is considered. These implications are crucial to the numerical descriptions of volcanic ash dispersal and deposition that are used to reduce volcanic risk to aviation and on the ground as well as for weather modeling that requires an accurate characterization of particulate matter in the atmosphere to provide reliable weather forecasting.

## Methods

### Evaluation of the rafting factor χ_R_

Particle rafting is evaluated comparing the terminal velocity of a single particle with respect to the settling velocity of the whole aggregate. The terminal velocity measured along the vertical axis *z* of a given object *i*—either aggregate (*i = A*) or single particle (*i = P*)—is defined as follows:

1 $$v_{ti} = \sqrt {\frac{4}{3}g\frac{{\left( {\rho _i - \rho _F} \right)}}{{\rho _F}}\frac{{d_i}}{{C_D}}}$$

where *ρ*_*i*_ and *d*_*i*_ are the density and diameter of the object, respectively; *ρ*_*f*_ is the air density, *g* is the acceleration due to gravity and *C*_*D*_ is the drag coefficient, a non-linear function of the particle Reynolds number *Re* which is ultimately dependent on the velocity *v*_*ti*_ itself. It is thus evident how Eq. (1) requires some iterative scheme to be solved.

An alternative approach to iterative schemes is to assume that the object is falling across an atmosphere characterized by a dynamic viscosity μ_*d*_. For simplicity, in the following we assume a still atmosphere, i.e., no wind is present in the environment. This assumption does not limit the generality of the analysis, as long as the particles and aggregates can locally reach their vertical terminal velocity. This condition is more affected by the physical properties of the atmosphere, such as its temperature, density and viscosity, more than by the presence or absence of a horizontal wind component. For this reason, we neglected the effect of wind on the determination of terminal velocities but, in the Supplementary Figs. 3–6 we presented additional sedimentation charts for a variety of atmospheric conditions.

The motion of such an object is ruled by Newton’s second law, where we assume that the external forces acting on the body are the gravitational force **F**_**g**_, the drag force **F**_**D**_ and the buoyancy force **F**_**b**_. Considering a downward positive orientation of the *z* axis (i.e. the resolution of the problem is along the vertical axis *z*, which means that the problem can be seen as one dimensional and the sign vector replaced with scalars), it reads as follows:

2 $$\frac{{d^2z}}{{dt^2}} = \frac{1}{{m_i}} \cdot \left( {F_g - F_D - F_b} \right)$$

where *F*_*g*_ = *m*_*i*_ *g*, and *F*_*b*_ = *V*_*i*_ *g*, denoting with *A*_*i*_ the projected area of the object along the direction of motion and *V*_*i*_ its volume. The terminal velocity of the object *v*_*ti*_ will be reached when the final acceleration of the body drops down an arbitrary small threshold *ε*. Equation (2) is solved numerically using a Runge-Kutta scheme of the 4–5^th^ order. The initial conditions for the object are *z*(*t* = 0) = 0 and *v*_*p*_(*t* = 0) = 0. The numerical solution of Eq. (2) is stopped when the difference between the velocities at time *i* and *i* + 1, respectively $v_p^i$ and $v_p^{i + 1}$, is less than *ε* = 10^−5^, i.e. $v_p^{i + 1} - v_p^i \,<\, \varepsilon$. This condition reveals that, within the selected threshold, the object is falling at its terminal velocity *v*_*ti*_ = *v*_*p*_.

For irregular objects described in terms of the form dimensions *L*,*I*,*S* (i.e. the axes derived from the maximum and the minimum area projection protocol^41,42^), it is convenient to express the diameter *d*_*p*_ as the geometric average *d*_*eq*_ = (*L I S*)^1/3^. In doing so, the object shape is approximated with its dimension-equivalent ellipsoid.

Replacing $F_g = \frac{\pi }{6}\rho _i\,g\,d_{eq}^3$, $F_D = \frac{1}{2}\rho _F\,C_D\frac{\pi }{4}d_{eq}^2\,v_i^2$ and $F_b = \frac{\pi }{6}\rho _F\,d_{eq}^3\,g$, the explicit form of Eq. (2) becomes:

3 $$\frac{{d^2z}}{{dt^2}} = g\left( {1 - \frac{{\rho _F}}{{\rho _i}}} \right) - \frac{3}{4}\frac{{\rho _F}}{{\rho _i}}\frac{{v_i^2}}{{d_{eq}}}C_D$$

For the determination of the Supplementary Fig. 2, Eq. (3) has been solved with two different formulations for the drag coefficient: the Stokes’ drag $C_D^{ST}$ and the drag $C_D^{BB}$ as reported for non-spherical particles:^20^

4 $$C_D^{ST} = 3\pi \,\mu _d\,d_{eq}\,v_i$$

5 $$C_D^{BB} = \frac{{24\,k_S}}{{Re}}\left[ {1 + 0.125\left( {\frac{{Re \cdot k_N}}{{k_S}}} \right)^{2/3}} \right] + \frac{{0.46\,k_N}}{{1 + \frac{{5330}}{{Re \cdot \frac{{k_N}}{{k_S}}}}}}$$

where *k*_*S*_ and *k*_*N*_ are respectively the Stokes and Newton correction terms that take into account the non-spherical shape of the falling object, defined as follows:

6 $$k_S = 0.5 \cdot \left( {F_S^{\frac{1}{3}} + F_S^{ - \frac{1}{3}}} \right)$$

7 $$k_N = 10^{0.45 \cdot [ - {\mathrm{log}}(F_N)]^{0.99}}$$

The parameters $F_S = f \cdot e^{1.3} \cdot \left( {\frac{{d_{eq}^3}}{{L\,I\,S}}} \right)$ and $F_N = f^2 \cdot e \cdot \left( {\frac{{d_{eq}^3}}{{L\,I\,S}}} \right)$ are the flatness $f = \frac{S}{I}$ and the elongation $= \frac{I}{L}$. In the work we assumed a spherical shape for falling aggregates, i.e. *k*_*S*_ = *k*_*N*_ = 1, as observed for cored clusters^17^; single particles are either assumed spherical or non-spherical according to the associated analysis. Eqs. (3) and (5) are solved numerically for a standard atmosphere at an altitude of 10,000 *m* a.s.l. with air density *ρ*_*F*_ = 0.46 kg m^−3^ and dynamic viscosity μ_*d*_ = 1.46∙10^−5^ Pa s.

Different values of air density and dynamic viscosities with respect to those shown in Fig. 2 are shown in Supplementary Figs. 3–6 for two different core densities. It is worth stressing that the terminal velocity of an aggregate strongly depends on its global density *ρ*_*A*_, which can be conveniently expressed as a function of the aggregate porosity $\phi _A = \frac{{V_{voids}}}{{V_A}}$:

8 $$\rho _A = \rho _P \cdot (1 - \phi _A)$$

where *V*_*voids*_ is the volume of all the voids in the aggregate, *V*_*A*_ is the aggregate volume and *ρ*_*P*_ is the density of each single component, here assumed constant at 2500 kg m^−3^ (Supplementary Note 1).

### Simulations with NAME

Particles used as aggregate cores in the Lagrangian simulations with NAME are derived from field analysis^23^. Two particular sizes are studied in the simulation: phi = 2 and phi = 3. We proceed evaluating those particles with sizes comprised within the bin limits under analysis and we evaluated the median of the equivalent diameters *D*_*eq*_ = (*LIS*)^1/3^. The two medians, one for each bin, identify two particles that will be used as a reference for the shape of particles belonging to phi = 2 and phi = 3. The median guarantees that the axes *L*, *I*, *S* used to characterize shapes in the simulation for phi = 2 and phi = 3 particles, really occurred during the observed explosive event. Other statistical descriptors, such as the mean, would have resulted in averaged values of *L*, *I*, *S*, that would not be representative of any of the collected ash. The details relative to NAME simulations discussed in sec. 2.3 are reported in Table 1.

## Supplementary information

Supplementary Information

## Data Availability

All data generated or analyzed during this study are included in this published article and its Supplementary files, and the codes available upon request from the authors. Since the work is mainly theoretical we accurately reported the equations and the procedure to reconstruct the figures in the Methods and Supplementary materials. Simulations with NAME can be reconstructed setting the initial conditions in Table 1 and Supplementary Note 4.
