# Supplementary material for: The fate of volcanic ash: premature or delayed sedimentation?
Source: Nat Commun. 2021 Feb 26;12:1303. doi: 10.1038/s41467-021-21568-8 (PMC7910302; doi:10.1038/s41467-021-21568-8)
Supplement: Supplementary file 1 — Supplementary Information [file 41467_2021_21568_MOESM1_ESM.pdf]

THE FATE OF VOLCANIC ASH: PREMATURE OR DELAYED SEDIMENTATION?

Supplementary Note 1 – Porosity, density and maximum porosity of a cored cluster (PC3)

The aim of this section is to calculate the aggregate porosity ϕ_A for cored clusters (see Supplementary Figure 1).

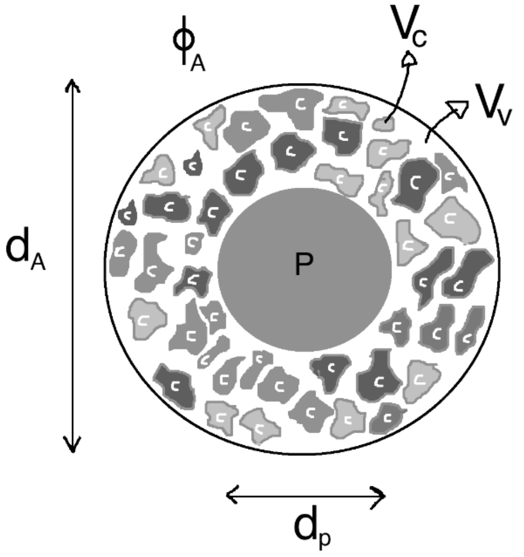

Supplementary Figure 1. Sketch of a cored cluster (PC3) with all the quantities introduced in the paper to derive the aggregate porosity ϕ_A. The "c" letter describes the coating particles. The letter "P" the inner core.

The aggregate porosity ϕ_A is defined as the ratio of all the voids V_{voids} and the total aggregate volume V_A:

$$\phi_A = \frac{V_{voids}}{V_A} = \frac{V_A - V_c - V_p}{V_A} = 1 - \frac{V_c}{V_A} - \frac{V_p}{V_A} \tag{1.1}$$

where V_c is the volume of ash coating and V_p the volume of the inner core. Notice how the definition of ϕ_A in Eq. 1.1 is formally different from the definition of porosity ϕ_A^L contained in Lane et al.[1], where $\phi_A^L = \frac{V_{voids}}{V_{coating}}$, where $V_{coating}$ is the volume of the coating part only.

The right-hand side of Eq. 1.1 can be rearranged as:

$$\phi_A = 1 - \frac{m_c \cdot \rho_A}{m_A \cdot \rho_c} - \frac{V_p}{V_A} \tag{1.2}$$

Eq. 1.1 can be rearranged as:

$$\frac{V_c}{V_A} = 1 - \phi_A - \frac{V_p}{V_A} \tag{1.3}$$

The overall aggregate density ρ_A can be expressed as a function of the coating mass m_c and the core mass m_p, regardless of the shape of the inner core:

$$\rho_A = \frac{m_A}{V_A} = \frac{m_p + m_c}{V_A} = \frac{(V_p \cdot \rho_p) + (V_c \cdot \rho_c)}{V_A} \qquad (1.4)$$

where ρ_c is the density of the ash coating, ρ_p is the density of the inner core and m_A the mass of the overall aggregate. Under the assumption that the coating and the inner core have similar densities, i.e. $\rho_c \approx \rho_p$, Eq.1.4 becomes:

$$\rho_A = \rho_p \cdot (\frac{V_p}{V_A} + \frac{V_c}{V_A}) \qquad (1.5)$$

Combining Eq.1.3 and Eq.1.5 we get an explicit relationship between the aggregate density and its porosity, as a function of ρ_p:

$$\rho_A = \rho_p \cdot \left(\frac{V_p}{V_A} + 1 - \phi_A - \frac{V_p}{V_A}\right) = \rho_p \cdot (1 - \phi_A) \qquad (1.6)$$

It is worth noticing that Eq.1.6 neither depends on the shape of the inner core nor is strictly related to cored clusters. Its validity is general, such as it can be easily proven defining the solid volume of the aggregate as $V_s = V_c + V_p$.
Let us evaluate the maximum aggregate porosity, noticing how Eq.1.2 provides the maximum aggregate porosity in the limit of an infinitesimal mass in the coating volume, i.e. for $m_c \to 0$.

$$\phi_A^{Max} = 1 - \frac{V_p}{V_A} \qquad (1.7)$$

Two cases are analysed in the following: i) spherical core; ii) ellipsoidal core.

Sphere: maximum aggregate porosity for an inner spherical core as a function of the aggregate-to-core size ratio Λ (i.e. $\Lambda = d_A/d_p$):

$$\phi_{A\_SP}^{Max} = 1 - \frac{V_p}{V_A} = 1 - \frac{\frac{\pi}{6}\, d_p^3}{\frac{\pi}{6}\, d_A^3} = 1 - \left(\frac{d_p}{d_A}\right)^3 = 1 - \frac{1}{\Lambda^3} \qquad (1.8)$$

Ellipsoid: maximum aggregate porosity for an inner ellipsoidal core as a function of the aggregate-to-core size ratios Λ_{LIS} (i.e. $\Lambda_{LIS} = d_A/d_{p_{eq}}$), where the three axes L, I, S are derived from the maximum and the minimum area projection[2]:

$$\phi_{A\_EL}^{Max} = 1 - \frac{V_p}{V_A} = 1 - \frac{\frac{\pi}{6}\,(L \cdot I \cdot S)}{\frac{\pi}{6}\, d_A^3} = 1 - \left(\frac{d_{p_{eq}}}{d_A}\right)^3 = 1 - \frac{1}{\Lambda_{LIS}^3} \qquad (1.9)$$

Therefore, the final formula for the maximum porosity has the same formal expression regardless of the shape of the inner core, once the diameter ratio α is properly defined.

Supplementary Note 2 – Comparison of the sedimentation charts for Stokes drag coefficient

In Supplementary Figure 2 we show the difference in rafting for different core sizes using Stokes' law, C_D^{ST} (Eq. 4 in methods) and Bagheri and Bonadonna's law[3] for drag, C_D^{BB} (Eq. 5 in methods).

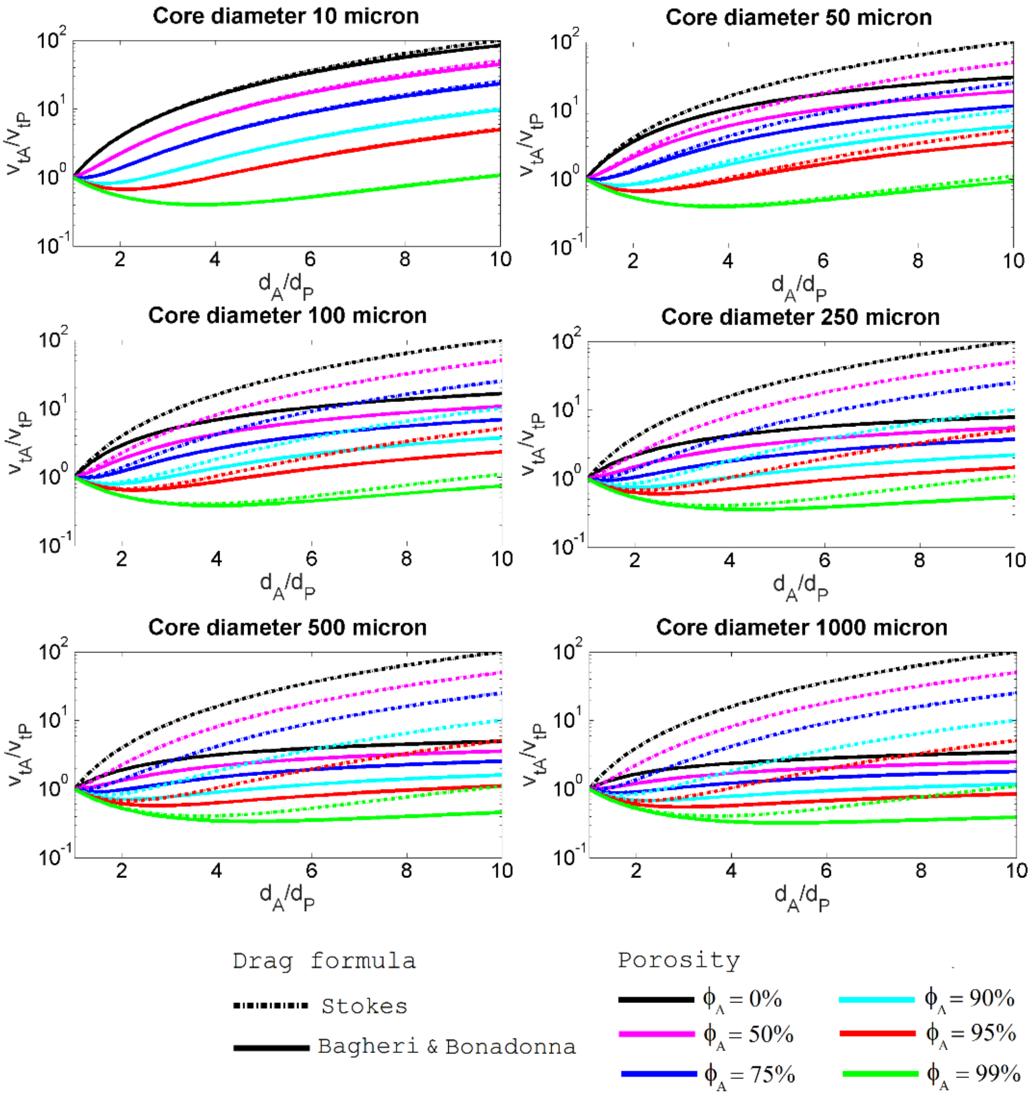

Supplementary Figure 2. Rafting parameter χ_R expressed as a function of $\Lambda = \frac{d_A}{d_P}$ and the aggregate porosity ϕ_A using two different formulations for the drag coefficient C_D: the Stokes' drag coefficient (dotted line) and Bagheri and Bonadonna (2016) (straight line).

Supplementary Note 3– Rafting as a function of core density and altitude of release

In this section the rafting parameter χ_R is calculated for core sizes from 10 μm to 1000 μm and densities of 2500 $\frac{kg}{m^3}$ and 1500 $\frac{kg}{m^3}$ respectively, for two different altitudes: 0 m a.s.l. (air density 1.22 $\frac{kg}{m^3}$ and dynamic viscosity $1.79 \cdot 10^{-5}\ Pa \cdot s$) and 20,000 m a.s.l. (air density 0.09 $\frac{kg}{m^3}$ and dynamic viscosity $1.42 \cdot 10^{-5}\ Pa \cdot s$).

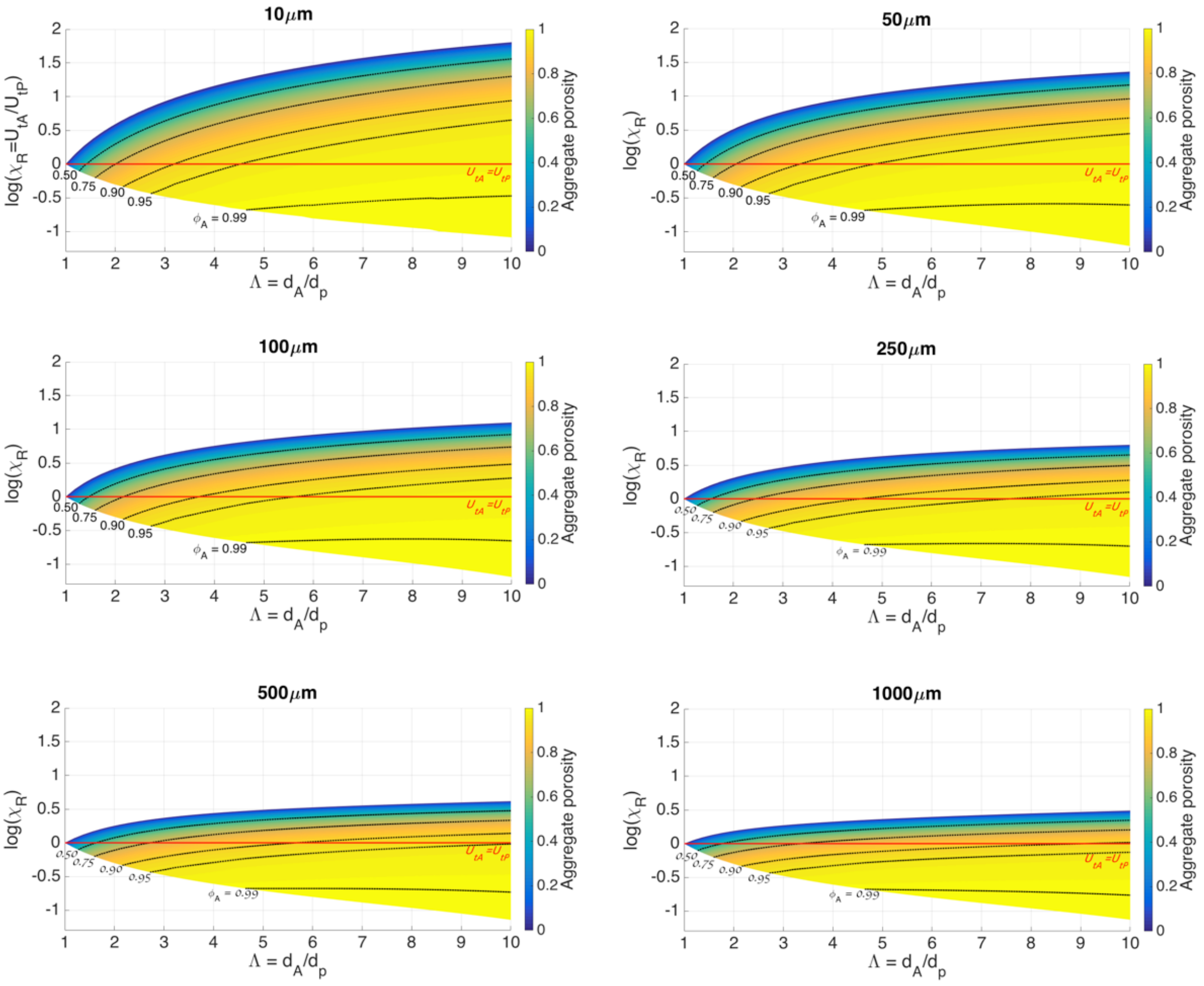

Supplementary Figure 3. Rafting parameter χ_R expressed as a function of $\Lambda = \frac{d_A}{d_P}$ for several values of aggregate porosity ϕ_A for a core density of 2500 $\frac{kg}{m^3}$, at 0 m a.s.l. .

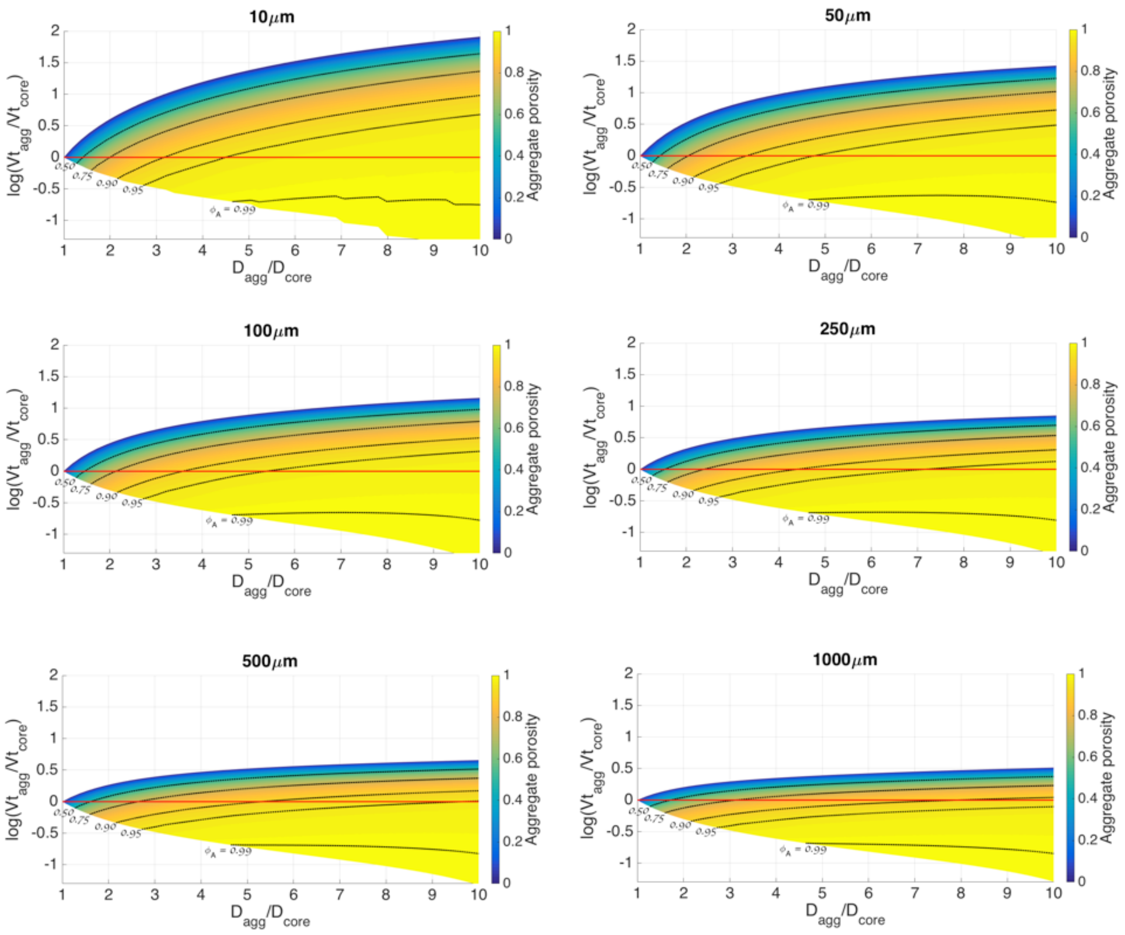

Supplementary Figure 4. Rafting parameter χ_R expressed as a function of $\Lambda = \frac{d_A}{d_P}$ for several values of aggregate porosity ϕ_A for a core density of 1500 $\frac{kg}{m^3}$ at 0 m a.s.l.

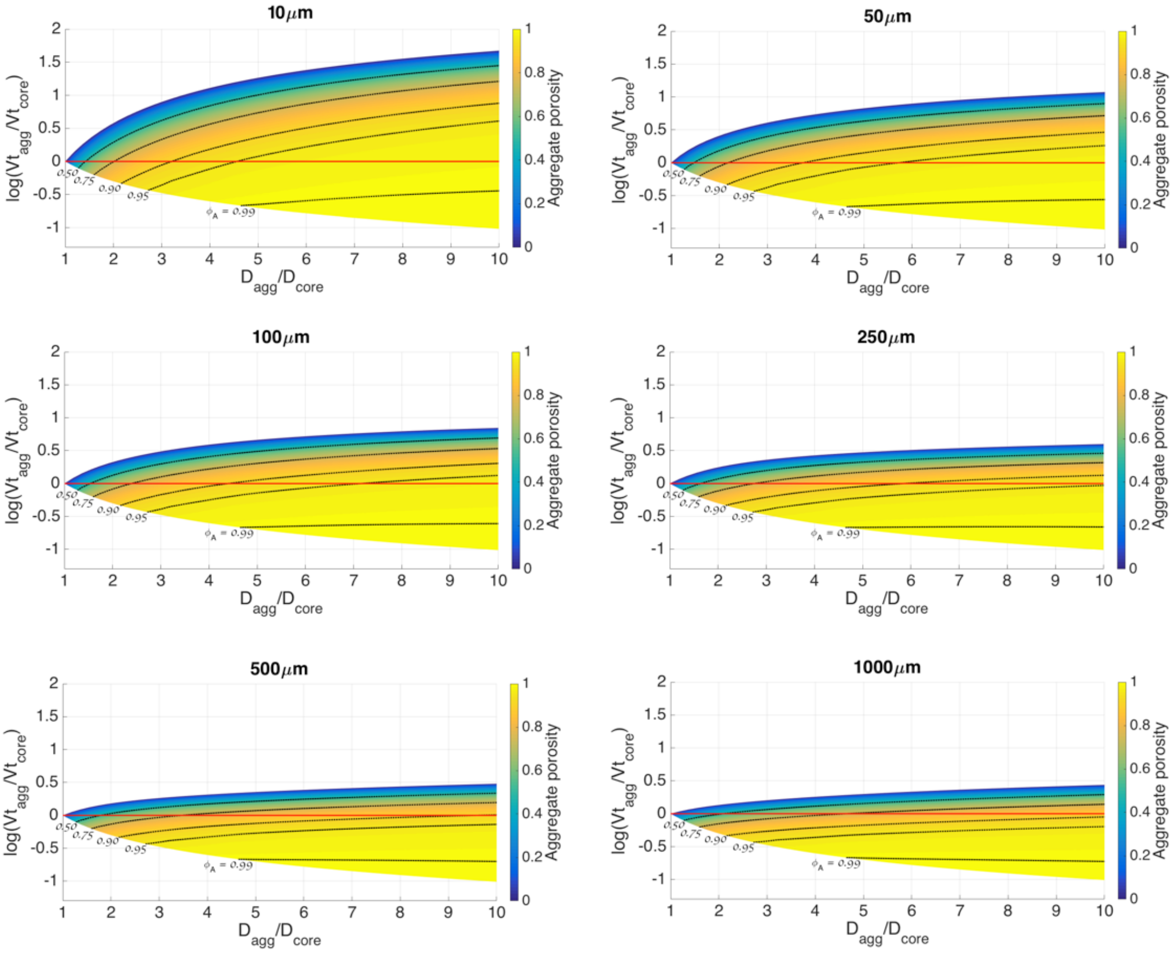

Supplementary Figure 5. Rafting parameter χ_R expressed as a function of $\Lambda = \frac{d_A}{d_P}$ for several values of aggregate porosity ϕ_A for a core density of 2500 $\frac{kg}{m^3}$, at 20,000 m a.s.l.

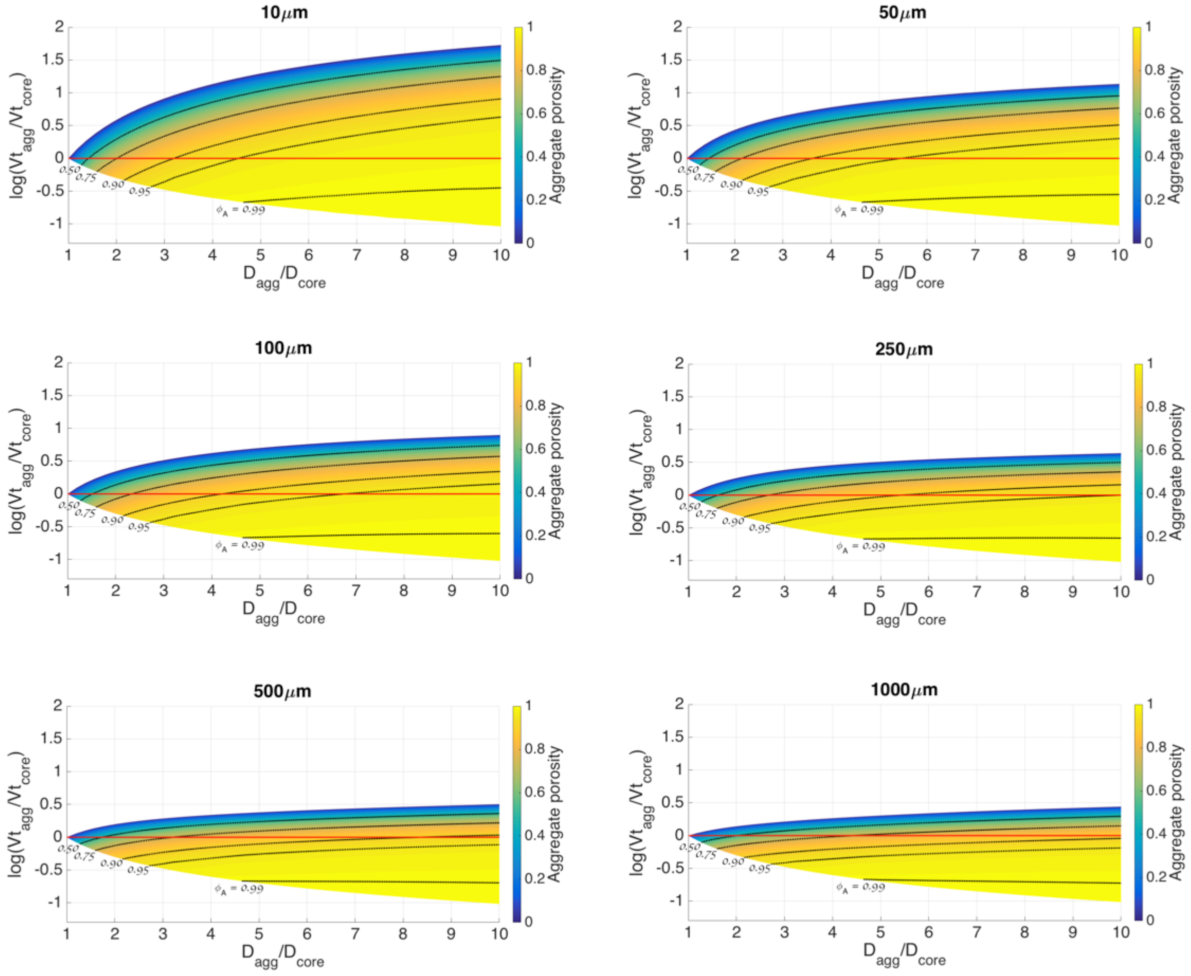

Supplementary Figure 6. Rafting parameter χ_R expressed as a function of $\Lambda = \frac{d_A}{d_P}$ for several values of aggregate porosity ϕ_A for a core density of 1500 $\frac{kg}{m^3}$, at 20,000 m a.s.l.

Supplementary Note 4 – Characteristics of aggregates and associated aggregate cores used as input data for Lagrangian simulations with NAME.

In the following tables we summarize the most important parameters used for the simulations in NAME. It is worth noticing that particle axes L, I, S are determined as defined in literature[3].

Supplementary Table 1. **Cores dynamical properties**. Shape factors and density values for single cores for ash collected at 9.7 km from the vent during the 2010 Eyjafjallajökull eruption (Iceland). The values reported in row 1 and row 3 belong to those particles whose diameter is equal to the median of D_{eq} for the two bins $\phi = 2$ and $\phi = 3$ respectively. The elongation and flatness are the ones proper of these two specific particles as observed for the Eyjafjallajökull ash[4]. In the simulation the statistical descriptor "median" guarantees the use of shape parameters that really occurred during the eruption. In row 2 and row 4 we reported the same particles but assumed spherical.

**Particle diameter D_{eq} [μm]** $D_{eq} = (L\,I\,S)^{\frac{1}{3}}$	**Particle diameter [ϕ] D_{eq}**	**Elongation (I/L)**	**Flatness (S/I)**	**Density [kg/m^3]**
138	2.85	0.72	0.70	2039
138	2.85	1	1	2039
447	1.16	0.84	0.76	1846
447	1.16	1	1	1846

Supplementary Table 2: **NAME runs for 138 μm particles**. Final combinations of particle diameters, density and sphericity applied in NAME simulation 1 (c.f. Figure 5a)

	Diameter D_{eq} (um)	Density (kg m-3)	Sphericity
Single Core Particle	138	2039	e =1 , f =1 (Spherical)
	138	2039	e = 0.89927, f = 0.88791
Aggregates	207	1223.4	e =1 , f =1 (Spherical)
	276	1223.4	e =1 , f =1 (Spherical)
	691	1223.4	e =1 , f =1 (Spherical)
	207	713.65	e =1 , f =1 (Spherical)
	276	713.65	e =1 , f =1 (Spherical)
	691	713.65	e =1 , f =1 (Spherical)
	207	203.9	e =1 , f =1 (Spherical)
	276	203.9	e =1 , f =1 (Spherical)
	691	203.9	e =1 , f =1 (Spherical)

Supplementary Table 3: **NAME runs for 447 µm particles.** Final combinations of particle diameters, density and sphericity applied in NAME simulation 2 (c.f. Figure 5b)

	Diameter D_{eq} (um)	Density (kg m-3)	Sphericity
Single Core Particle	447	1864	e =1 , f =1 (Spherical)
	447	1864	e = 0.84029, f = 0.75594
Aggregates	670	1118.4	e =1 , f =1 (Spherical)
	894	1118.4	e =1 , f =1 (Spherical)
	1341	1118.4	e =1 , f =1 (Spherical)
	670	652.4	e =1 , f =1 (Spherical)
	894	652.4	e =1 , f =1 (Spherical)
	1341	652.4	e =1 , f =1 (Spherical)
	670	186.4	e =1 , f =1 (Spherical)
	894	186.4	e =1 , f =1 (Spherical)
	1341	186.4	e =1 , f =1 (Spherical)

Supplementary Note 5 – Table of constants

Symbol	***Meaning***	***Units***
Re	Reynolds number	*[-]*
ρ_F	Air density	$\frac{kg}{m^3}$
d_P	Single particle diameter	m
ρ_P	Single particle density	$\frac{kg}{m^3}$
v_{tA}	Aggregate terminal velocity	m/s
v_{tP}	Single particle terminal velocity	m/s
$\chi_R = \frac{v_{tA}}{v_{tP}}$	Rafting factor	*[-]*
v_{ti}	Terminal velocity of the *i*[th] object	m/s
ρ_i	Density of the *i*[th] object	$\frac{kg}{m^3}$
d_i	Diameter of the *i*[th] object	m
g	Acceleration of gravity	m/s^2
C_D	Drag coefficient	*[-]*
C_D^{ST}	Stokes drag coefficient	*[-]*
C_D^{BB}	Bagheri and Bonadonna (2016) drag coefficient	*[-]*
k_S	Stokes correction term for the shape	*[-]*
k_N	Newton correction term for the shape	*[-]*
ϕ_A	Aggregate porosity	%
V_{voids}	Volume of the internal voids in an aggregate	m^3
V_A	External volume of the aggregate	m^3
μ_d	Dynamic viscosity	$Pa \cdot s$
ρ_A	Aggregate density	$\frac{kg}{m^3}$
$\Lambda = \frac{d_A}{d_P}$	Aggregate-to-particle diameters ratio	*[-]*
ϕ-scale	Krumbein scale	*[-]*
$d_{eq} = (L \cdot I \cdot S)^{1/3}$	Equivalent diameter calculated as the geometric mean of the three axes	m
L	Largest axis measured from the maximum projected area	m
I	Smallest axis measured from the maximum projected area	m
S	Smallest axis measured from the minimum projected area	m
F_S	Stokes form factor	*[-]*
F_N	Newton form factor	*[-]*
f	Flatness	*[-]*
e	Elongation	*[-]*

Supplementary References

1. Lane S.J., Gilbert J.S., Hilton M. The aerodynamic behavior of volcanic aggregates. Bulletin of Volcanology 55, 481-488 (1993).

2. Bagheri G.H., Bonadonna C., Manzella I., Vonlanthen P. On the characterization of size and shape of irregular particles. Powder Technol. 270, 141-153 (2015).

3. Bagheri G., Bonadonna C. On the drag of freely falling non-spherical particles. Powder Technol. 301, 526-544 (2016).

4. Bonadonna C., et al. Tephra sedimentation during the 2010 Eyjafjallajokull eruption (Iceland) from deposit, radar, and satellite observations. J. Geophys. Res-Solid Earth 116, 20 (2011).
